# Factors influencing insulin acceptance among type 2 diabetes mellitus patients in a primary care clinic: a qualitative exploration

**DOI:** 10.1186/1471-2296-14-164

**Published:** 2013-10-29

**Authors:** Hasliza Abu Hassan, Hizlinda Tohid, Rahmah Mohd Amin, Mohamed Badrulnizam Long Bidin, Leelavathi Muthupalaniappen, Khairani Omar

**Affiliations:** 1 Primary Care Medicine Discipline, Universiti Teknologi MARA (UiTM), Shah Alam, Selangor 40450, Malaysia; 2 Department of Family Medicine, Universiti Kebangsaan Malaysia Medical Centre, JalanYaacob Latiff, Bandar Tun Razak, 56000 Cheras, Kuala Lumpur, Malaysia; 3 Faculty of Medicine and Health Science, Universiti Sultan Zainal Abidin (UniSZA), Kota Campus, Jalan Sultan Mahmud, Kuala Terengganu, Terengganu 20400, Malaysia; 4 Endocrinology Unit, Department of Medicine, Kuala Lumpur General Hospital, 50586 Kuala Lumpur, Malaysia; 5 Department of Family Medicine, Universiti Sains Islam Malaysia, 71800 Nilai, Negeri Sembilan, Malaysia

**Keywords:** Type 2 diabetes mellitus, Insulin, Insulin resistance, Qualitative research

## Abstract

**Background:**

Many Type 2 Diabetes Mellitus (T2DM) patients refuse insulin therapy even when they require this modality of treatment. However, some eventually accept insulin. This study aimed to explore the T2DM patients’ reasons for accepting insulin therapy and their initial barriers to use insulin.

**Methods:**

This qualitative study interviewed twenty-one T2DM patients at a primary care clinic who had been on insulin for more than a year through three in-depth interviews and three focus group discussions. A semi structured interview protocol was used and the sessions were audio-recorded. Subsequently, thematic analysis was conducted to identify major themes.

**Results:**

The participants’ acceptance of insulin was influenced by their concerns and beliefs about diabetes and insulin. Concerns about complications of poorly controlled diabetes and side effects of other treatment regime had resulted in insulin acceptance among the participants. They also had a strong belief in insulin benefits and effectiveness. These concerns and beliefs were the results of having good knowledge about the diabetes and insulin, experiential learning, as well as doctors’ practical and emotional support that helped them to accept insulin therapy and become efficient in self-care management. These factors also allayed their negative concerns and beliefs towards diabetes and insulin, which were their barriers for insulin acceptance as it caused fear to use insulin. These negative concerns were related to injection (self-injection, needle phobia, injection pain), and insulin use (inconvenience, embarrassment, lifestyle restriction, negative social stigma, and poor self-efficacy), whereas the negative beliefs were 'insulin could cause organ damage’, 'their diabetes was not serious enough’, 'insulin is for life-long’, and 'insulin is for more severe disease only’.

**Conclusions:**

Exploring patients’ concerns and beliefs about diabetes and insulin is crucial to assist physicians in delivering patient-centered care. By understanding this, physicians could address their concerns with aim to modify their patients’ misconceptions towards insulin therapy. In addition, continuous educations as well as practical and emotional support from others were found to be valuable for insulin acceptance.

**Trial registration:**

Universiti Kebangsaan Malaysia FF-214-2009.

## Background

Early use of insulin in the management of poorly controlled diabetes has been recommended to prevent and reduce the long-term diabetes complications [1,2]. It reduces patients’ exposure to prolonged hyperglycemia, which ultimately increases risks of diabetes-related complications [3]. However, delay in insulin initiation is common. About 50% of patients with poor control T2DM did not timely start insulin therapy and the initiation was usually three to five years after failure of oral hypoglycemic agents [4,5]. There are many factors influencing delayed insulin initiation including those caused by healthcare providers and its system, as well as the patients themselves [6-8]. One of the main barriers is psychological insulin resistance (PIR), defined as psychological opposition towards insulin use, among patients and healthcare providers [7,9,10].

About 27% of the UKPDS patients allocated to insulin therapy was found to have refused insulin [11]. Even among insulin-naïve diabetes patients in the Western community, a comparable proportion (28-39%) was reluctant to be on insulin [12-14]. However, higher proportion of PIR was reported by Asian studies, quoting prevalence between 51–70.6% [15,16].

Gherman et al. (2011) had reviewed 60 literatures on PIR and summarised factors for PIR into four main categories [10]: (1) emotional factors (e.g. fear of injection pain and needle, apprehension of self-injection, fear of injection technique or correct dosing, and fear of consequences of insulin use, such as hypoglycaemia, weight gain, lifestyle restriction, and inconvenience) [13-15,17,18], (2) cognitive factors (e.g. perception of poor self-efficacy, personal failure or ineffectiveness of insulin, belief that own diabetes is not serious enough, and insulin is for more severe diabetes) [15,17-20], (3) social/cultural factors (e.g. social stigma and embarrassment) [15,20,21], and (4) relational factors (e.g. influence by others particularly health care providers) [18,22,23]. Physical factors (e.g. pain or bruising due to injection) also cause insulin refusal among those who have agreed to take insulin, resulting in omission or skipping of insulin [13,15,17,18].

In general, PIR has been extensively examined through reviews, perspectives in practice, editorials, as well as quantitative and qualitative studies [10]. Based on previous PIR studies, the insulin-naïve diabetes patients who were more willing to accept insulin therapy were males, and those with tertiary education, insulin-using relatives, more diabetes-related complications, strong self-efficacy and better relationship with their healthcare providers [14-16,23]. They have more positive perceptions about insulin in term of its effectiveness in improving their glycemic control and general health, as well as preventing diabetes complications [14-16,23]. Woundenberg et al. (2012) also found that these patients were less likely to oppose to lifelong insulin therapy, which was related to their confidence in beneficial effects of insulin [14].

Perceiving insulin as beneficial is a crucial factor for diabetes patients commencing on insulin to accept insulin therapy [24,25]. It is influenced by how important good glycemic control to them and their confidence that insulin could help them achieving it [25]. Their expectation and understanding that good glycemic control improves their health and well being also shape their perception [25,26]. As described by Morris et al. (2005), diabetes patients identified insulin as a 'friend’ or 'foe’ and this influenced their coping with the therapy [24]. Experience of using insulin over time had helped them to rationalise the benefits and accept insulin as 'a friend’ [24]. The experience empowered their confidence to use insulin by learning that they were able to injecting themselves and adjusting the insulin dose [24]. It allowed demonstration of insulin efficacy through achieving better glycemic control and well beings, thus validating their perception of insulin benefits [24].

On-going experience with treatments through experimentation and discussions with healthcare providers has been found to influence diabetes patients in deciding what works for them [26]. They did not consciously assess benefits and risks of a treatment at the point of its commencement; instead their treatment decision-making was a continuous process [26]. In a study by Phillips (2007), the participants who had been taking insulin for at least one year described that their experience was far less traumatic than what they had expected [27]. Many of them felt healthier after insulin initiation [27]. They were able to cope with insulin use and could manage their diabetes well [27].

Due to limited number of studies exploring diabetes patients’ experience taking insulin, our understanding about how and why patients accept insulin is still unclear. Many studies have focused on the patients’ difficulties taking insulin, their coping mechanism and perceptions on insulin. Furthermore, the studies were carried out in western countries that commonly practice independent individualism culture. In contrast to Asian countries, which have more dependent collectivism culture. These different types of culture play an important role in influencing diabetes patients’ belief and attitude towards their health and health behaviour. Studying the factors influencing insulin acceptance is crucial to formulate effective strategies for insulin initiation. A qualitative approach allows detailed exploration of experience, feelings, beliefs and attitude of the diabetes patients in the context of insulin acceptance. Thus, this study aimed to explore factors that influence T2DM patients in Malaysia to accept insulin. Their barriers towards insulin use which they experienced prior insulin initiation was also explored.

## Methods

This qualitative study involved twenty-one T2DM patients who had been on insulin for one year or more (Table 1). The participants were purposively selected from those who had been attending a primary care clinic in Kuala Lumpur from February 2009 to January 2010. Patients who were assumed to be able to generate rich, thick and meaningful information based on their experience, reminiscence and ability were invited to participate in this study. They were from different age groups, ethnicity, gender, and socioeconomic status. They also had various duration of diabetes and duration of insulin use. This was to ensure heterogeneity of the data. Patients with history of serious medical condition at the time of insulin initiation and those who could not communicate in either Bahasa Malaysia or English were excluded. The sample size of the study was determined when data saturation was reached. This was taken as the point at which redundancy of information occurred or when no more new information was expected to emerge [28].

**Table 1:** The characteristics of the participants

	**In depth interview**	**Focus group discussion**
	**(IDI 1, 2 & 3)**	**(FGD 1, 2 & 3)**
	**(n = 3)**	**(n = 18)**
**Age (year)**	58–67	40–68
**Ethnicity**		
Malay	1	10
Chinese	-	4
Indian	2	4
**Gender**		
Female	1	7
Male	2	11
Educational status*		
Primary level	1	8
Secondary level	1	5
Tertiary level	1	5
**Duration of diabetes (year)**	8–13	5–14
**Duration of insulin use (year)**	3–4	2–6
**Occupations**	Retiree, lecturer, housewife	Housewife, chef, police officer, government officer, retired businessman, security officer, teacher, retired nurse, clerk, retired army, retired contractor, insurance agent, businessman, engineer, driver

*Educational status: Primary = schooling from age 7–12 years old.

Secondary = schooling up to age 17 years old.

Tertiary = schooling up to college or university level.

After obtaining the informed consents, the participants were interviewed either individually (in-depth interviews (IDIs)) or in groups (focus group discussions (FGDs)) (Table 1). All interviews were moderated by the main researcher, a postgraduate student of family medicine who received training in qualitative research. The interviews were carried out at the primary care clinic where the participants received their care. Educational status of the diabetes patients had been the basis of sampling in this study as it was hypothesised to influence their attitude towards insulin. Furthermore, grouping the patients for FGD according to their educational status could facilitate data generation. To optimise rigour of the study, discussions with both homogeneous and heterogeneous focus groups in term of educational status were performed. Participants with similar educational status would feel less intimidated to share their experience and opinion and this could allow in-depth discussion of issues. Therefore, in this study, homogeneity of a group was ensured in two of the FGDs. However, using this recruitment approach could result in biased findings and limited information gained from such groups [29]. Since this study was exploratory in nature, maximising range of opinion was crucial. Therefore, participants with various educational status were grouped in one of the FGDs to allow generation of rich data. The session was moderated by a trained personnel to assist in free sharing of opinion and experience in a heterogeneous group.

Each interview was conducted over one and a half hour, using a semi-structured interview protocol (Table 2). Many of the participants were bilingual, hence they were allowed to converse in Bahasa Malaysia or English, depending on their preference. A standard protocol was developed to explore the reasons for acceptance of insulin. The participants were allowed to discuss their feelings and perception regarding insulin when its use was first recommended. During the interview, the participants’ responses to the main questions (as in the protocol) were followed to expand their comments and explore the issues in depth through probing, rephrasing, and clarifying. All the interview sessions were audio and video recorded. The video recording was necessary to assist in identifying the participants in the audio-recordings.

**Table 2:** Interview protocol used in the in-depth interviews and group discussions

**Main issues**	**Questions**
Initial feelings when insulin was first suggested	Question 1: How did you feel when you were first told to start on insulin therapy? Explore
Initial perception or belief about insulin	Question 2: What was your perception about insulin at that time? Explore
Factors influencing insulin acceptance	Question 3: Why did you accept insulin therapy? Explore
Perception or belief about insulin when accepting insulin	Question 4: What was your perception about insulin at the time when you agree to use insulin? Explore

After each interview, the audio recording was transcribed and the transcript was examined against the actual recordings to ensure accuracy. Thematic analysis was performed using NVIVO 7 (QSR International Pty Ltd, Victoria, AU) to identify themes found in each transcript. Eventually, all themes were categorised into three major themes. These were: (1) participants’ feelings towards insulin use, (2) their perception about insulin, and (3) their reasons for accepting insulin. To ensure the reliability and accuracy of the coding process, two other researchers crosschecked the coding of the data.

### Ethical issues, reliability, and validity

This study received ethical approval from the Research and Ethic Committee of the Universiti Kebangsaan Malaysia and permission to perform the study at the University Primary Care Clinic was obtained. In addition, all participants provided written consents prior to their interviews and their identities were preserved to ensure confidentiality.

To ensure reliability and validity of the study, several strategies were implemented: (1) triangulation of data collection through three IDIs and three FGDs, (2) self-reflectivity and peer review or debriefing, (3) procedural validity through proper interview technique, and (4) cross-checking of the coding process in the thematic analysis.

## Results

### Sociodemographic characteristics of the participants

Twenty-one T2DM patients were interviewed through three in-depth interviews (IDIs) and three focus group discussions (FGDs). Data saturation was reached during the last three interviews (two IDI and one FGD).

There were 8 female and 13 male participants; 11 Malays, 4 Chinese and 6 Indians. Their age ranged between 40 to 68 years old (Table 1). The average duration of having diabetes was 8 years and the average duration of insulin use was 3.2 years.

### Participants’ feelings when insulin use was first recommended

Many participants had negative concerns related to insulin use. The main concerns vented by them were about injection of insulin:

(1) Painful injection.

“I am scared of needle.. you know, the poking itself, it is painful.. using needle some more, and you poke yourself.. it is painful” (3 years of insulin use/ 6 years of having diabetes).

“Injection is painful” (3 years of insulin use/ 6 years of having diabetes).

(2) Apprehension about self-injection.

“I could not do it myself. I could not poke myself like that. The fear of injecting myself when the doctor asked me to use insulin.. Urgghhh.. I dared not” (2.5 years of insulin use/ 6 years of having diabetes).

“I never had anything against insulin but I could not seeing myself to inject insulin” (4 years of insulin use/ 10 years of having diabetes).

(3) Needle phobia.

“I have phobia of needles. Last time, they used quite a long needle.” (3 years of insulin use/ 6 years of having diabetes).

(4) Social embarrassment.

“Sometimes it is embarrassing to inject insulin in public places like restaurants.” (6 years of insulin use/ 14 years of having diabetes).

“It is not easy when people watch you injecting insulin. It is embarrassing” (2 years of insulin use/ 5 years of having diabetes).

Apart from concerns related to injection, the participants were worried about the impacts of using insulin:

(1) Inconvenience and impractical.

“I see half an hour before dinner she (my daughter-in law’s mother) has to inject.. I said..so troublesome, very inconvenient” (2.5 years of insulin use/ 6 years of having diabetes).

“I don’t think using needle is practical” (3 years of insulin use/ 6 years of having diabetes).

(2) Lifestyle restriction.

“When you are on insulin, it is more difficult to go out or eat out. You are more restricted. You have to plan your exercise as well” (6 years of insulin use/ 10 years of having diabetes).

(3) Social stigma.

“Our society is quite ignorant of insulin therapy and they might associate insulin injection with drug addicts” (2 years of insulin use/ 5 years of having diabetes).

In addition, they had concerns with their own ability to manage insulin use and one participant claimed it was due to poor counselling by the HCP:

“I was not confident to handle the injection, use the needle, the timing… I was not confident” (4 years of insulin use/ 10 years of having diabetes).

“When I was first prescribed insulin, I was not counselled properly. Nobody gave me proper explanation on how and when to administer insulin, how to look after insulin pens and how to overcome hypoglycaemia. I had some doubts and was not very confident to start insulin therapy” (4 years of insulin use/ 10 years of having diabetes).

#### Participants’ perception about insulin when it was first recommended

The participants in this study admitted to have certain perception about insulin that resulted in their reluctance to use insulin. They believed that:

(1) Insulin causes organ damage.

“When the doctor prescribed insulin, I was terrified of detrimental effects of insulin on kidneys. I was afraid that insulin might ruin my health” (3 years of insulin use/ 5 years of having diabetes).

(2) Insulin is not indicated as their diabetes is regarded as not serious enough.

“When I first heard of insulin, I was made to understand that your disease is very serious when you need insulin. That was why I refused insulin initially. I did not want to be in a very serious category” (3 years of insulin use/ 8 years of having diabetes).

(3) Insulin is only for severe diabetes.

“My friends and siblings told me that people who received insulin were very serious. They might die soon” (3 years of insulin use/ 5 years of having diabetes).

(4) Insulin is for life-long.

“Because I know insulin is something that you have to live with for the rest of your life. You cannot go off insulin” (2 years of insulin use/ 8 years of having diabetes)

#### Impacts of the participants’ initial feelings and perception on insulin use

Many participants were reluctant to use insulin when their doctors first recommended insulin. Their concerns and negative perception about insulin had resulted them to have fear to use insulin:

“I was actually scared of insulin because it requires injection” (2 years of insulin use/ 10 years of having diabetes).

“I was scared of insulin injection” (2 years of insulin use/ 5 years of having diabetes).

### Factors for insulin acceptance

Similarly as described by the participants for their initial insulin resistance, their concerns and beliefs about diabetes and insulin had played important factors in their treatment decision-making. In this study, the participants admitted that their concerns about the complications of poorly controlled diabetes and side effects of other treatment regime made them to accept insulin:

“I agreed to take insulin after the doctor informed me that high blood sugar could cause stroke and kidney failure. So when I heard about the risk of having a stroke, I was scared.” (3 years of insulin use/ 10 years of having diabetes).

“Furthermore, the (oral) medications can cause kidney damage” (5 years of insulin use/ 10 years of having diabetes).

Their beliefs about insulin and other diabetes treatment had triggered them to accept insulin use, including:

(1) Insulin is effective treatment to improve their health, maintain good glycemic control or prevent complications.

“I know insulin makes you healthier and more energetic. That’s why I took it” (3 years of insulin use/ 12 years of having diabetes).

“I know insulin is good. Sugar control becomes better and you have better quality of life. At the end of the day, you just want to have a good life, right… not just physical, but your mental and soul as well” (2.5 years of insulin use/ 6 years of having diabetes).

“To me, insulin is good for our health because it can control blood sugar and prevent amputation and dialysis” (2 years of insulin use/ 5 years of having diabetes).

(2) Insulin is natural.

“I know that insulin is very natural to the body. That is why (this) information is very important to ease the acceptance to insulin therapy” (3 years of insulin use/ 8 years of having diabetes).

(3) Limited benefits of oral hypoglycemic agents.

“I accepted insulin because the pills were not effective anymore. Pills are not effective in long standing diabetes, not effective in controlling diabetes… not effective anymore” (6 years of insulin use/ 10 years of having diabetes).

“I know after a while, oral medication cannot bring down blood sugar. I took insulin because the pills were not working anymore” (5 years of insulin use/ 2 years of having diabetes).

The participants claimed that their subsequent positive attitude towards insulin use was a result of having good knowledge about diabetes and its treatment, obtained through reading, surfing internet and their relatives:

“I gained a lot of knowledge from self-reading and relatives who are on insulin” (2 years of insulin use/ 5 years of having diabetes).

“I read a lot about insulin, I surf internet. That’s why I know about the benefits of insulin” (3 years of insulin use/ 8 years of having diabetes).

“I always refer to these two 'specialists’ (my father and older brother who are on insulin) when it comes to insulin” (6 years of insulin use/ 10 years of having diabetes).

The participants also admitted to accept insulin through learning about benefits of insulin from other people’s experience (experiential learning):

“After I saw my husband had better sugar control with insulin, I accepted insulin” (5 years of insulin use/ 2 years of having diabetes).

“How could I refuse insulin? My father and older brother are on insulin. My brother’s health was better after he took insulin.” (6 years of insulin use/ 10 years of having diabetes).

They affirmed that their acceptance became stronger as they personally experienced the benefits of insulin in terms of improved general health, well-being and self-care behaviour after they started using the insulin:

“It feels different to be on insulin. I feel better when I use insulin. I used to have blurred vision before using insulin. Now I have good vision” (3 years of insulin use/ 8 years of having diabetes).

“When I was on oral treatment, I was always sleepy and tired. After taking insulin, I become more energetic and more confident in life” (3 years of insulin use/ 12 years of having diabetes).

“When I’m on insulin, I became more concerned of my health. I will check my sugar two or three times per day. I notice that the sugar will rise if I do not take insulin. I become more careful with food intake and do more exercise” (5 years of insulin use/ 2 years of having diabetes).

Furthermore, the participants admitted that good relationship with their doctors had prepared them to use insulin. Effective communication (e.g. polite and clear explanation), relationship with respect and trust, as well as patient-centred counselling, were specifically mentioned by the participants as their reasons to accept insulin:

“When the doctor advised me to start insulin therapy, I felt embarrassed to reject his recommendation. The doctor was 'cool’, never angry; talked nicely and that made me feel so comfortable. After such thorough explanation, any patient will be embarrassed to say no to insulin.” (4 years of insulin use/ 8 years of having diabetes).

“When the doctor advised me to start on insulin, the explanation was clear” (2 years of insulin use/ 5 years of having diabetes).

“You accept the insulin because the bond is there and you respect him (doctor)” (4 years of insulin use/ 8 years of having diabetes).

“I accepted insulin because I listened to the doctor. The doctor advised me to take insulin as it can improve my blood sugar. Whatever he (doctor) says, I will follow because I trust him” (3 years of insulin use/ 6 years of having diabetes).

“..we discussed about the issues of insulin, my worries and thoughts about insulin. I became less apprehensive and was ready to start on insulin therapy” (2 years of insulin use/ 5 years of having diabetes).

## Discussion

Generally, both positive and negative concerns and beliefs about diabetes and insulin were the basis of the participants’ decision to use insulin in this study. Initially, many participants were reluctant to use insulin as a result of their negative concerns about insulin injection. This anxiety related to injection (i.e. fear of injection pain or needle) is actually very common among diabetics, reported as high as 71% among insulin naive diabetics [13,15,16,24,30,31]. Morris et al. (2005) highlighted possible relation between needle phobia and the needle size [24]. Thus, use of thinner and shorter needles could reduce this fear, making injection less painful. However, Snoek et al. (2007) found that a substantial proportion of diabetes patients who were treated with insulin (38%) using thinner needles still admitted “Injecting insulin is painful” [31]. This proportion was not far from those who were insulin naïve (43%), though the difference was found significant [31]. Despite this finding, a striking drop in fear of injection (from 47% among insulin naïve patients to 6% among insulin-treated patients) was observed [31]. Perhaps, pain anticipated by the insulin naïve patients may be exaggerated compared to the real pain experienced by those who are using insulin. It is tolerable than they initially expected [27]. This could explain the drop in fear of insulin injection despite painful injection. The other reason for fear of injection is apprehension of self-injection, which was common (40%) among insulin naïve patients [13,27]. As insulin injection was found less traumatic than the patients initially anticipated, this apprehension would diminish with time resulting in lesser fear of insulin injection [27].

Embarrassment of injection reported by the participants in this study was also found significant by Snoek et al. (2007), in which 23% of the insulin naïve patients admitted that injecting insulin was embarrassing as compared to 10% of insulin-treated patients [31]. Due to difference in culture, more insulin naïve patients in Malaysia (55.9%) described such embarrassment [15]. In the study by Nur Azmiah et al. (2011), 64.9% of insulin naïve patients who were not willing to be on insulin perceived 'injecting is embarrassing', which was significantly higher than those who were willing (46.7%) [15].

Having wrongly identified as drug addicts by others is another concern of embarrassment shared by the participants. It is a common worry among insulin naïve diabetics and it is related to the use of syringes and vials [7,21], which is still common in Malaysia.

In this study, concerns about the impacts of insulin use on the participants’ life also made them refused insulin in the first place. These worries were substantiated as almost 50% of diabetes patients on insulin felt that its use restricted their life [12,15,18]. In fact, one third of insulin users had difficulties in fulfilling their work and personal responsibilities [16]. Wong et al. (2011) suggested that this factor could reflect their lower self-efficacy [16], which was also reported by the participants in this current study.

As injection-related concerns were prevalent among diabetics, this should be evaluated and addressed appropriately by healthcare providers. Many diabetes patients prefer more effective insulin with fewer side effects (e.g. insulin analogues) and new insulin-delivery devices (e.g. prefilled insulin pens with shorter needles) that are less invasive and more convenient [32,33]. This insulin has been found to provide greater lifestyle flexibility, reduce social stigma and needle phobia, as well as improve self-efficacy and compliance [32,34-37]. Thus, its use should always be considered to alleviate injection-related concerns.

Distorted belief and misconceptions about insulin held by the participants in the current study were similar to previous studies [13,15,38]. These studies found increased PIR if the patients believed that insulin could worsen diabetes or cause complications [13,15]. This is similar to our participants’ belief that insulin could cause organ damage. They also thought insulin was for severe diabetes and they were in denial regarding their diabetes control, which was similarly found in Funnel (2008) [38]. However, Nur Azmiah et al. (2011) found that, as the patients’ perceived disease severity increased, their likelihood for PIR increased as well [15]. This is contrary to the Health Belief Model that posits higher tendency for patients to change their health behaviour (in this context is to use insulin) if they believe they have more severe disease [39]. These misconceptions could reflect the participants’ poor knowledge regarding diabetes and insulin when they were first recommended for insulin [9]. A qualitative study done in Malaysia by Mohd Ali and Jusoff (2009) found that majority of their diabetes patients lacked understanding about diabetes, its management, and the effects of treatment [40]. Thus, early education at the point of diagnosis regarding the progressive nature of diabetes and inevitable use of insulin could allay their misconceptions [9,10]. Therefore, healthcare providers must be prepared with adequate information and counselling skill to ensure the delivery of diabetic education is effective and individualised to the patients’ needs [41].

After initiation of insulin, the participants’ active effort to seek for knowledge on diabetes and its treatment might contribute to their insulin acceptance. Their good level of knowledge may be responsible for their renewed concerns and beliefs that favour insulin use [14,23]. Better understanding about the disease could also equip patients with higher self-efficacy to manage their diabetes [40]. As there is various knowledge-seeking methods found to be practiced by the participants in this study, easy access to information related to diabetes and its treatment should be provided through multiple strategies (e.g. one-stop centre, website or pamphlets) to enhance insulin acceptance. This support can be provided by trained nurses and it can reduce time constrain to initiate insulin experienced by physicians [41].

Experiential learning through observing others coping with insulin use and having benefits of insulin had helped the participants to alleviate their negative concerns and misconceptions about insulin. Support from relatives and friends who used insulin had promoted their insulin acceptance. These findings were concurrent with Hunt et al. (1997) showed that previous personal experience, observations and interaction with other influence ones’ attitude towards insulin [22]. Perhaps, the aforementioned factors were the reason why those with more insulin-using relatives were more likely to accept insulin [14,23]. In view of these, peer support group may be effective to promote insulin acceptance among insulin naive diabetics as peer testimonies could facilitate insulin initiation and intensification [10,41].

Apart from these, the participants’ personal experience using insulin had resulted in strong belief in insulin benefits and self-efficacy in using insulin. They experienced at first-hand the benefits of insulin in improving their health, well being, and their glycemic control, as similarly found in Morris et al. (2005) and Phillips (2007) [24,27]. Perhaps, they also thought that insulin use was less traumatic then they initially anticipated as claimed by the diabetes patients in Phillips (2007) [27]. As a result, the experience helped them to continue insulin use despite having some reluctance in the beginning [24,26]. In fact, those who had experienced using insulin would have improved self-efficacy and better understanding about diabetes [27]. Therefore, 'trial of insulin’ can be promoted to diabetes patients to provide them the opportunity to gain such positive experience. Before the trial, demonstration of insulin device, its needle and injection technique should be provided [42]. During the demonstration, healthcare providers should allow the patients to touch the device and try injecting themselves with placebo [42].

Apart from the participants’ experience in using insulin, practical and emotional support from physicians had helped them to accept insulin and become more self-efficient to use insulin. These findings emphasise the importance of good, trust and respect relationship between patients and healthcare providers. This relationship was claimed achievable through effective communication and patient-centred approach. Good interaction and interpersonal relationship between patients and their healthcare providers had been found to be the main promoters for insulin use in previous studies [22,23,26]. In addition, easy access support and quality relationship with the healthcare providers could increase patients’ knowledge, self-efficacy, greater general well-being, lesser diabetes-related distress, and better adherence to self-care activities (lifestyle and medication regimens) [43]. However, dominance of healthcare providers in making decision to initiate insulin for their patients, as observed in Phillip (2007) and Morris et al. (2005) [24,27], should be avoided. This is because informing patients about their treatment options and involving them in making treatment decisions could improve their adherence to the opted treatment [43]. Thus, regular training in effective communication for healthcare providers is undeniably crucial.

Limitations of this study are mostly related to its design. This study involved only those from an urban clinic, thus generalisation of findings should be done cautiously. In addition, recall bias might be possible as selected participants were those who had been on insulin at least for more than a year. For future study, exploring ambivalence to use insulin among those who have been recently initiated insulin by their healthcare providers is recommended. This could provide further information about their decision making process.

## Conclusions

Patients’ concerns and beliefs regarding insulin use are very much influenced by their knowledge, experience and support from others. Thus it is crucial to provide them with knowledge (through effective communication, support and educational activities), as well as positive experience for them to adopt favourable concerns and beliefs that promote insulin acceptance. In addition, delivering patient-centred care and good doctor-patient relationship will contribute to achieving long term treatment targets to both the patient and physician.

## Abbreviations

T2DM: Type 2 diabetes mellitus; PIR: Psychological insulin resistance; HCP: Healthcare providers; IDI: In-depth interview; FGD: Focus group discussion.

## Competing interest

The authors declare that they have no competing interests.

## Authors’ contributions

Conception and design, analysis and interpretation of the data: HAH, RA, KO, HT. Obtaining of funding: HAH, KO. Provision of study materials, collection and assembly of the data: HAH. Drafting and critical revision of the article: HT. Final approval of the article: HT, HAH, RA, MBLB, KO, LM. Administrative, technical, or logistic support: RA, KO. All authors read and approved the final manuscript.

## Pre-publication history

The pre-publication history for this paper can be accessed here:

http://www.biomedcentral.com/1471-2296/14/164/prepub
